# Cytokine, Chemokine, and Metalloprotease Activation in the Serum of Patients with Nephropathia Epidemica from the Republic of Tatarstan and the Republic of Mordovia, Russia

**DOI:** 10.3390/pathogens10050527

**Published:** 2021-04-27

**Authors:** Ekaterina Martynova, Yuriy Davidyuk, Emmanuel Kabwe, Ekaterina E. Garanina, Venera Shakirova, Vera Pavelkina, Yulia Uskova, Robert J. Stott, Toshana L. Foster, Maria Markelova, Mehendi Goyal, Abhimat Gupta, Mannan Bhola, Vinay Kumar, Manoj Baranwal, Albert A. Rizvanov, Svetlana F. Khaiboullina

**Affiliations:** 1 Institute of Fundamental Medicine and Biology, Kazan Federal University, 420008 Kazan, Russia; davi.djuk@mail.ru (Y.D.); emmanuelkabwe@ymail.com (E.K.); kathryn.cherenkova@gmail.com (E.E.G.); mimarkelova@gmail.com (M.M.); rizvanov@gmail.com (A.A.R.); sv.khaiboullina@gmail.com (S.F.K.); 2 Infectious Diseases Department, Kazan State Medical Academy, 420012 Kazan, Russia; vene-shakirova@yandex.ru; 3 Infectious Diseases Department, National Research Ogarev Mordovia State University, 430005 Saransk, Russia; pavelkina@rambler.ru (V.P.); juliamurzilka@gmail.com (Y.U.); 4 Faculty of Medicine and Health Sciences, School of Veterinary Medicine and Science, Sutton Bonington Campus, The University of Nottingham, Loughborough LE12 5RD, UK; robert.stott@nottingham.ac.uk (R.J.S.); toshana.foster@nottingham.ac.uk (T.L.F.); 5 Doconvid.ai, Bestech Business Tower, Mohali 160055, India; goyalmehendi700@gmail.com; 6 Department of Computer Science and Engineering, Thapar Institute of Engineering and Technology, Patiala 147004, India; abhimatg0004@gmail.com; 7 Department of Biotechnology, Thapar Institute of Engineering and Technology, Patiala 147004, India; docmab23@gmail.com (M.B.); manoj.baranwal@thapar.edu (M.B.); 8 Department of Electronics and Communication Engineering, Thapar Institute of Engineering and Technology, Patiala 147004, India; vinay.kumar@thapar.edu

**Keywords:** Nephropathia Epidemica, cytokine, chemokine, metalloprotease

## Abstract

Nephropathia Epidemica (NE), endemic to several Volga regions of Russia, including the Republic of Tatarstan (RT) and the Republic of Mordovia (RM), is a mild form of hemorrhagic fever with renal syndrome caused by infection with rodent-borne orthohantaviruses. Although NE cases have been reported for decades, little is known about the hantavirus strains associated with human infection in these regions. There is also limited understanding of the pathogenesis of NE in the RT and the RM. To address these knowledge gaps, we conducted comparative analyses of patients with NE in the RT and the RM. Clinical symptoms were more severe in patients with NE from the RM with longer observed duration of fever symptoms and hospitalization. Analysis of patient sera showed changes in the levels of numerous cytokines, chemokines, and matrix metalloproteases (MMPs) in patients with NE from both the RT and the RM, suggesting leukocyte activation, extracellular matrix degradation, and leukocyte chemotaxis. Interestingly, levels of several cytokines were distinctly different between patients NE from the RT when compared with those from the RM. These differences were not related to the genetic variation of orthohantaviruses circulating in those regions, as sequence analysis showed that Puumala virus (PUUV) was the causative agent of NE in these regions. Additionally, only the “Russia” (RUS) genetic lineage of PUUV was detected in the serum samples of patients with NE from both the RT and the RM. We therefore conclude that differences in serum cytokine, chemokine, and MMP levels between the RT and the RM are related to environmental factors and lifestyle differences that influence individual immune responses to orthohantavirus infection.

## 1. Introduction

Orthohantaviruses are enveloped negative-sense single-stranded RNA viruses, belonging to the family *Bunyaviridae*, that are the causative agents of hemorrhagic fever with renal syndrome (HFRS). HFRS is typically characterized by fever, increased vascular permeability, thrombocytopenia, and acute kidney injury, and currently it has the highest incidence rate of all zoonotic virus infections reported in Russia [1,2]. Nephropathia Epidemica (NE) is a mild form of HFRS that is endemic in the Volga Federal District of Russia, that includes the Republic of Tatarstan (RT) and the Republic of Mordovia (RM) [3,4,5,6]. Mortality rate is low (0–0.4%) and complete recovery of kidney function without development of chronic renal disease or acute kidney injury (AKI) is commonly expected [7,8,9]. Still, NE presents a significant public health concern due to the high incidence of infection and lack of specific treatment [10,11]. NE is caused by the Puumala virus (PUUV), a member of the *orthohantavirus* genus, found circulating persistently yet asymptomatically within reservoir populations of bank voles (*Myodes glareolus*) [12,13]. Human infection is believed to occur via inhalation of virus-contaminated aerosols of bank vole excreta and secreta [14]. Upon infection, the virus disseminates and primarily targets endothelial cells, with little-to-no documented cytopathic impact on the endothelium associated with virus replication [15,16]. Therefore, the severity of viral pathogenesis and disease progression is believed to be largely due to the contribution of host immune response factors and mechanisms that are activated during viral infection.

Studies have shown that clinical presentation may differ in patients infected with the same strain of orthohantavirus, with a high proportion of PUUV infections reported to be subclinical and many cases remaining undiagnosed [17]. Additionally, single-nucleotide mutations in pro-inflammatory cytokine tumor necrosis factor (TNF)-α have been reported to coincide with increased severity of PUUV-induced NE [18]. Case-fatality rates for PUUV-induced NE have previously been reported to rise with age, and female patients were observed to have a higher mortality rate in the first year after diagnosis with acute NE; inferring that age and sex may represent predictive variables of clinical outcome [19]. The geographical, endemically active region has also been shown to influence the severity of orthohantavirus infections; Klempa and colleagues reported that the severity of Dobrava virus (DOBV) cases was higher in the district of Sochi in southern Russia when compared with that of Kurkino DOBV cases in the central Russian district of Lipetsk [20]. Although many biological markers have been suggested to determine orthohantavirus fatality and disease severity [21,22,23], the mechanisms defining the differences observed in clinical presentation remain largely unknown.

Clinical characteristics of NE in the RT and the RM are observed to be similar, with the onset of disease coinciding with acute, flu-like symptoms [24]. NE cases were characterized with symptoms of lumbar pain and decreased urinary output, varying from anuria and oliguria to polyuria. [25,26]. In some cases, the urinary output can remain unaffected with only initial transient proteinuria being reported [27]. AKI is associated with the most severe form of NE/HFRS. Hemorrhages, varying from small petechiae to severe internal bleeding, are often reported in all stages of the disease [28,29]. Laboratory results typically reveal thrombocytopenia, proteinuria, creatininemia, and uremia [30]. Increased serum levels of chemokines and proinflammatory cytokines are also documented in patients with NE; we have previously shown upregulation of serum levels of interferon (IFN)-γ, interleukin (IL)-10, CCL2, and IL-12 in NE cases from the RT when compared to those of controls [31]. Activation of regulatory cytokines such as CCL2, CCL3, and CXCL10 has also been reported in serum samples of patients with NE from the Republic of Bashkortostan (RB) [32]. The cytokine storm hypothesis is suggested to be responsible for orthohantavirus pathogenesis, where increased endothelial permeability and kidney dysfunction is due to the upregulation of proinflammatory cytokines that, too, may determine the severity of clinical presentations and disease. Data from our previous studies support this hypothesis, with increased serum levels of cytokines consistently observed in NE cases from the RT and the RB regions of Russia [31,32].

Although NE is endemic in the RM [6], human immune responses to orthohantavirus infection remain largely un-characterized. In addition, details of the cytokine responses to hantavirus infection in patients with NE from the RM is largely unknown. Therefore, in this present study we determined the orthohantavirus strains associated with NE in the RM and analyzed the cytokine activation in patients with NE from the RM. Additionally, comparative analyses were conducted to characterize orthohantavirus strains associated with NE cases from the RM and the RT.

## 2. Materials and Methods

Patients. Serum samples were collected from 58 NE cases (49 male and 9 female; average age 38.77 (37.9, 43.44)) admitted to the Republican Infectious Disease Clinical Hospital, Saransk, the RM and 98 NE cases (72 male and 26 female; mean age 41 (39.13, 46.2)) admitted to the Agafonov Republican Clinical Hospital for Infectious Disease, the RT. All samples were collected during the acute phase of the disease at the time of the admission and at the convalescent phase during the discharge. Diagnosis of NE was established based on clinical presentation and was serologically confirmed by detection of anti-orthohantavirus IgM antibodies. Serum samples from 27 control individuals from the RM (12 male and 15 female; age 29.8 to 30.8) and 30 control individuals from the RT (18 male and 13 female; age 34.1 to 31.7) were collected.

Multiplex Analysis. Serum cytokine levels were analyzed using Bio-Plex (Bio-Rad, Hercules, CA, USA) multiplex magnetic bead-based antibody detection kits following the manufacturer’s instructions. Multiplex kits, Bio Plex Pro Human Cytokine 27-plex Panel, Bio Plex Human Cytokine 21-plex Panels, and Bio Plex Human Matrix metalloproteases (MMPs) were used for detection of a total of 84 cytokines. Serum aliquots (50 μL) were analyzed where a minimum of 50 beads per analyte was acquired. Median fluorescence intensities were collected using a Luminex 100 or 200 analyzer (Luminex, Austin, TX, USA). Each sample was analyzed in triplicate. The collected data were analyzed with MasterPlex CT control software and MasterPlex QT analysis software (MiraiBio, San Bruno, CA, USA). Standard curves for each cytokine were generated using the standards provided by the manufacturer.

RT-PCR detection of PUUV transcripts. Total RNA was extracted from serum using TRIzol Reagent (Invitrogen Life Technologies^TM^, Carlsbad, CA, USA) following the manufacturer’s recommendations. cDNA was synthesized using Thermo Scientific RevertAid Reverse Transcriptase (Thermo Fisher Scientific, Waltham, MA, USA). Nested PCR was carried out using TaqPol polymerase (Sileks, Badenweiler, Germany). Primers are summarized in Table 1. The resulting PCR products of 19 PUUV strains from the RT and 8 PUUV strains from the RM were purified with Isolate II PCR and Gel Kit (Bioline, London, UK) and subsequently sequenced using ABI PRISM 310 big Dye Terminator 3.1 sequencing kit (ABI, Vernon, CA, USA). Sequences were deposited in the GenBank database under accession no. MW587790-MW587800, MW587805-MW587819, MW587821.

Phylogenetic analysis. Phylogenetic analysis of PUUV partial S segment sequences was performed using MEGA v6.0 software [34]. Nucleotide sequences of 13 PUUV strains obtained in the bank vole populations in the RT and 5 strains from GenBank were used (Accession No: PUUV/Observatory/MG_118/2015, MW587801; PUUV/Vysokaya Gora/MG_1388/2018/, MW587802; PUUV/Kazan/MG_845/2017, MW587804; PUUV/Lenino-Kokushkino/MG_1140/2017, MW504252; PUUV/Laishevo/MG_809/2017, MW504247; PUUV/Teteevo/MG_1041/2017, MT495382; PUUV/Mamadysh/MG_980/2017, MW504250; PUUV/Naberezhnye Chelny/MG_260/2015, MW504226; PUUV/Naberezhnye Chelny/MG_260/2015, MW504226; PUUV/Krasnyi Klyuch/MG_158/2015, MW504225; PUUV/Tatarskoe Utiashkino/MG_1419/2019, MW504213; PUUV/Starye Salmany/MG_1589/2019, MW504240; PUUV/Lesnye Morkvashi/MG_794/2017, MW587803; Puu/Kazan, Z84204; Samara_49/CG/2005, AB433843; DTK/Ufa-97, AB297665; Sotkamo2009, HE801633; PUUV/Orleans/Mg29/2010, KT247595), Tula orthohantavirus strain Sennickerode Sen05/205, EU439951 was used as an outgroup. Phylogenetic trees were constructed using the maximum parsimony method included in Mega v6.0 [34]. The bootstrap values calculated for 1000 replicates are given as percentages and the values less than 70% are not shown.

Hantavirus ELISA. Detection of anti-orthohantavirus antibodies was used as a confirmatory test for NE diagnosis [34,35]. The VektoHanta IgG ELISA kit and Vector Hanta IgM kit (Vektor Best, Novosibirsk, Russia) were used to determine hantavirus-specific antibodies [34,35]. Briefly, the serum from patients with NE and controls was diluted 1:100 (PBS) and incubated for 60 min at 37 °C in a 96-well plate with pre-adsorbed hantavirus antigens. Following washing (3×; 0.5% Tween20 in PBS, PBS-T), wells were incubated with anti-human-IgG-HRP or anti-human-IgM-HRP conjugated antibodies (1:10,000 in PBS-T, Amerixan Qualex Technologies, San Clemente, CA, USA) for 30 min at 37 °C. Post incubation and washing (3×; 0.5% Tween20 in PBS), wells were incubated with 3,3′,5,5′ Tetramethylbenzidine (Chema Medica, Moscow, Russia). The reaction was stopped by adding an equal amount of 10% phosphoric acid (TatKhimProduct, Kazan, Russia). Data were measured using a microplate reader Tecan 200 (Tecan, Männedorf, Switzerland) at OD_450_ with reference OD_650_. OD_450_ values higher than 0.5 were considered to be positive results.

Statistical analysis. Statistical analysis was performed in the R environment [36]. Statistically significant differences between groups of patients in different stages and control volunteers were accepted as *p* < 0.05, assessed by the Kruskal–Wallis test with Benjamini–Hochberg adjustment for independent populations and Wilcoxon signed rank test for paired data.

## 3. Results

Clinical presentation of NE cases from the RT and the RM. A total of 25 NE cases (21 male, 4 female) from the RM and 98 NE cases (72 male, 26 female) from the RT were included in this study (Table 2). The average age of patients was similar in both regions (37.28 ± 13.64 years in the RM and 41 ± 15.22 years in the RT). NE diagnosis was based on clinical presentation, epidemiological data, and serological confirmation (Table 2). Many clinical symptoms of the disease in both regions were similar; however, patients with NE from the RM had a significantly longer hospitalization period (15.4 ± 1.0 days) when compared to patients with NE in the RT (9.9 ± 3.6 days). In addition, the number of days with fever was longer in patients from the RM (7.8 ± 1.9 days) than in patients from the RT (5.4 ± 1.9 days). These data suggest that the clinical presentation of NE differs in the two endemically active regions.

**Figure 1:**
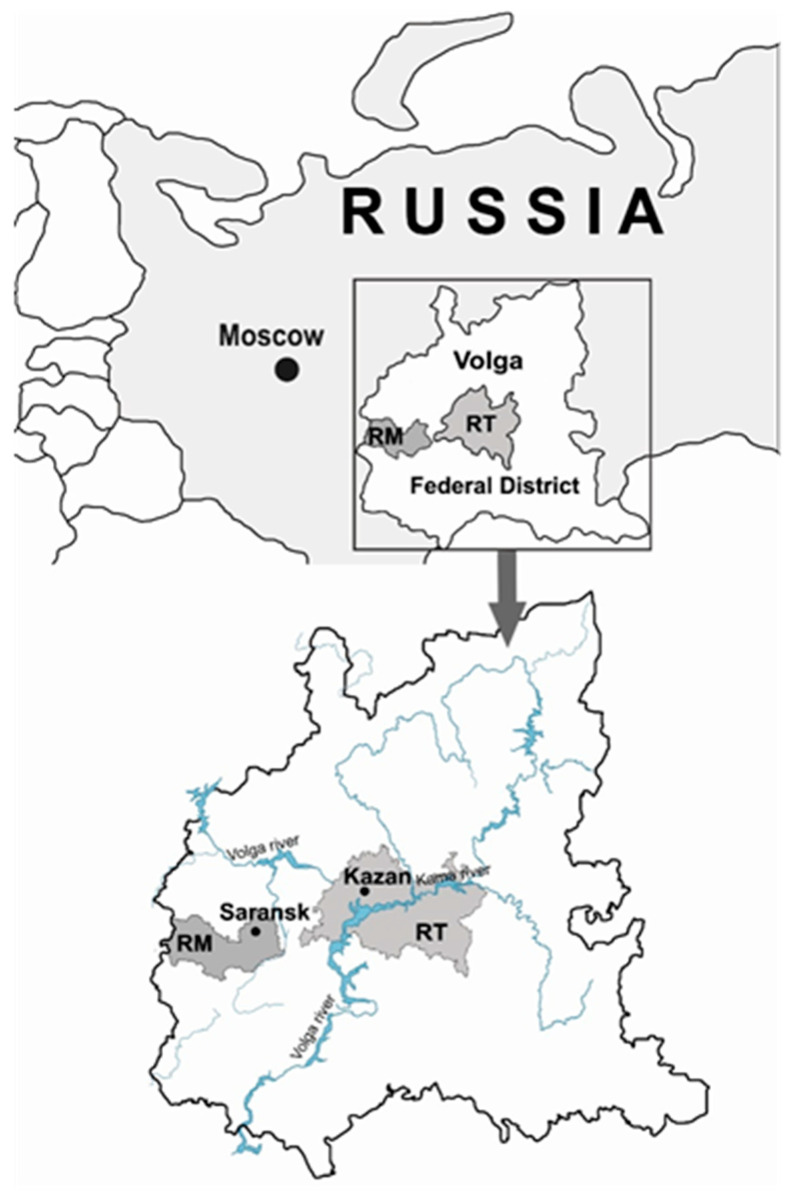
Map of Volga Federal District. Dark grey—the RM with capital Saransk; Light Grey—the RT with capital Kazan.

Phylogenetic analysis of PUUV identified in NE cases from the RT and the RM. The RT and the RM are part of the Volga Federal District, which is endemic for NE (Figure 1) [3,4,5,6].

**Figure 2:**
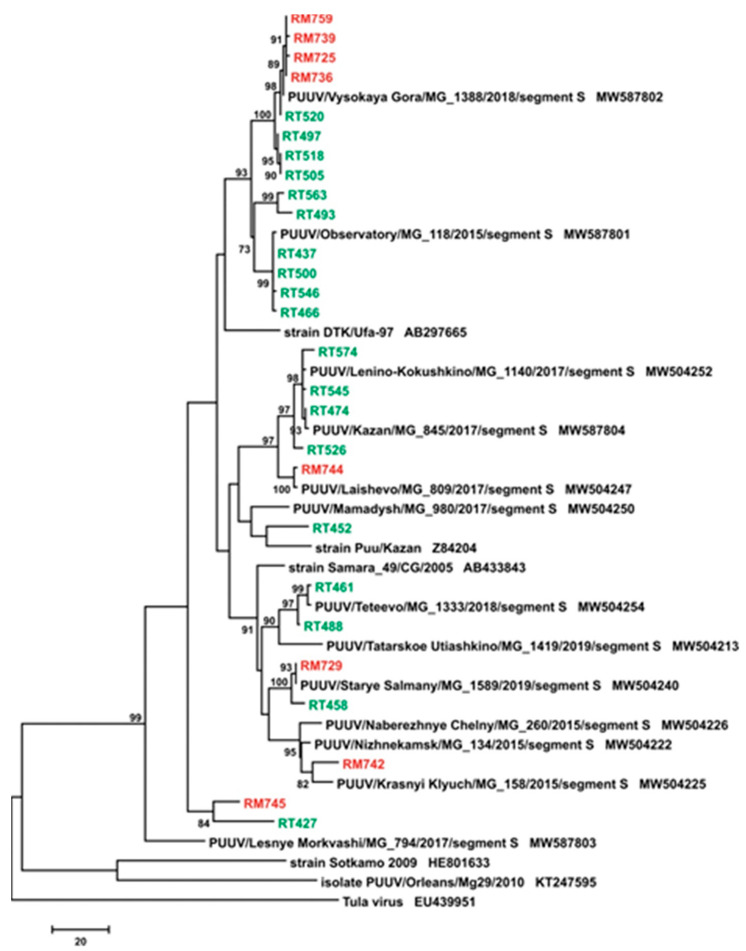
Phylogenetic analysis of PUUV S segment sequences from the RT (green) and the RM (red). Phylogenetic tree for the partial S segment (640 bp long, nt 705–1344 based on GenBank sequence Z84204) of PUUV strains from the RT and the RM generated using the maximum parsimony method. The percentage of replicate trees in which the associated taxa clustered together in the bootstrap test (1000 replicates) are shown next to the branches (65) only values greater than 70% are shown. The MP tree was obtained using the subtree-pruning-regrafting (SPR) algorithm (66).

Two orthohantaviruses, PUUV and DOBV, have previously been detected in HFRS cases documented in the Volga Federal District [37,38]. It appears that DOBV is the etiological agent of severe HFRS, whilst PUUV causes NE, a mild-to-moderate form of HFRS [10]. However, Tula virus (TULV), which has been isolated from common voles, is believed to be non-pathogenic in humans [39]. Thus, given the differences in the hospitalization period and duration of fever in patients from the RT and the RM, we sought to determine whether these contrasts could be explained by the infection of patients with different orthohantaviruses. PCR products from the 19 RT and 8 RM NE cases were therefore sequenced and were determined as variations of PUUV. DOBV and TULV sequences were not detected. Sequence analysis of viral RNA revealed that all obtained strains belonged to the Russian genetic lineage of PUUV and were phylogenetically related to PUUV strains circulating in bank vole populations in the RT (Figure 2).

We therefore identified PUUV as the causative infectious agent of NE from the RT and the RM. These data support previous observations that PUUV is the prevalent etiological agent of NE in the Volga Federal District [38,40,41], which includes the RT, the RM, the RB, and Udmurtia. Therefore, we suggest that differences in cytokine activation in NE from the RT and the RM could be explained by host immune response mechanisms to viral infection.

Analysis of serum cytokine and MMP levels in controls from the RT vs. the RM. Regional differences in patient serum cytokines have been demonstrated in multiple infectious diseases [42,43,44,45,46], suggesting a role for cytokine activation in inflammation severity. Therefore, we sought to determine whether serum cytokine and MMPs levels differ in NE from the RM and the RT. We have found that serum levels of IL-1α, IL-6, IL-7, b-NGF, GM-CSF, and MMP13 were significantly different, while levels of the remaining 78 cytokines and MMPs were not significantly different between regional controls (Supplementary Materials Table S1).

**Figure 3:**
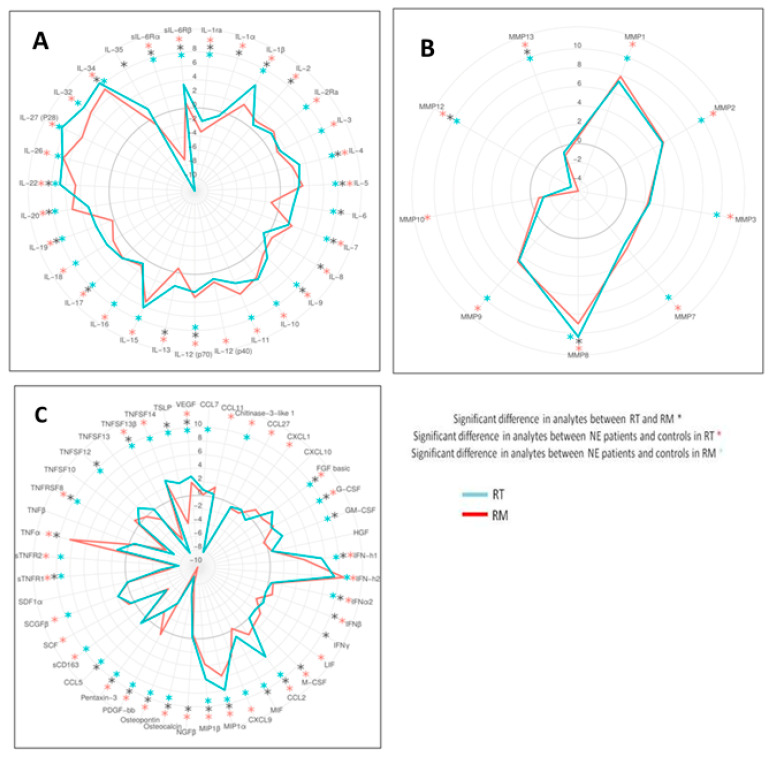
Comparison of analytes in serum of patients with NE from the RT and the RM. Serum samples from acute patients with NE in the RT and the RM were analyzed and the levels of (**A**) interleukins, (**B**) matrix metalloproteases (MMPs), and (**C**) cytokines were compared with corresponding regional controls. Data are presented as Log_2_ fold changes relative to regional controls. Kruskal–Wallis test with Benjamin–Hochberg adjustment were used to identify statistical significance (*p* < 0.05) and a significant difference in analytes was found between the RT and the RM (black asterisks); significant difference in analytes between patients with NE and controls in the RT (red asterisks); significant difference in analytes between patients with NE and controls in the RM (blue asterisks).

Analysis of acute NE-induced changes in cytokine and MMP levels from the RT and the RM. A total of 84 cytokines and MMPs were analyzed in serum from control and patients with NE from the RT and the RM (Figure 3; summarized in Supplementary Materials Table S2).

We found that levels of the majority of cytokines and MMPs were significantly altered in patients with NE from the RT and the RM when compared to that of the local controls (Figure 3, red and blue stars). Levels of 46 cytokines and eight MMPs were significantly altered in serum from patients with NE from both the RT and the RM (Figure 3, both red and blue stars). Of these, 52 shared a similar trend in patients from both the RT and the RM when compared with respective controls, suggesting that disease pathogenesis and physiological responses are comparable in both regions. Levels of IL-1α, IL-2, IL-8, IL-12(p40), IL-13, MMP10, CCL11, CCL27, CXCL1, IFNβ, LIF, M-CSF, NGFβ, SCF, and TNFα were altered only in the RT when compared with those of controls (Figure 3; red stars only), while IL-6, CCL7, CCL5, GM-CSF, MIF, TNFSF12, and TSLP were altered only in the RM NE samples compared to those of local controls (Figure 3, blue stars only). Together, 52 out of 84 cytokines and MMPs studied possessed a similar trend in NE from the RT and the RM suggesting that the pathogenesis of the disease in both regions shares many similarities.

Although multiple cytokines and MMPs differ similarly compared to regional controls, the degree of changes vary in NE from the RT and the RM. We found that levels of 30 cytokines were significantly higher in patients with NE from the RM than in those from the RT, while 15 cytokines were lower in acute patients with NE from the RM compared to those of patients in the RT (Figure 3A,C; black stars). In addition, we found that patients with NE from the RT had higher levels of MMP8 than did patients with NE from the RM, but lower levels of MMP12 and MMP13 (Figure 3B; black stars). Therefore, we suggest that, although appearing similar, some aspects of NE pathogenesis in the RT differ from that in the RM.

**Figure 4:**
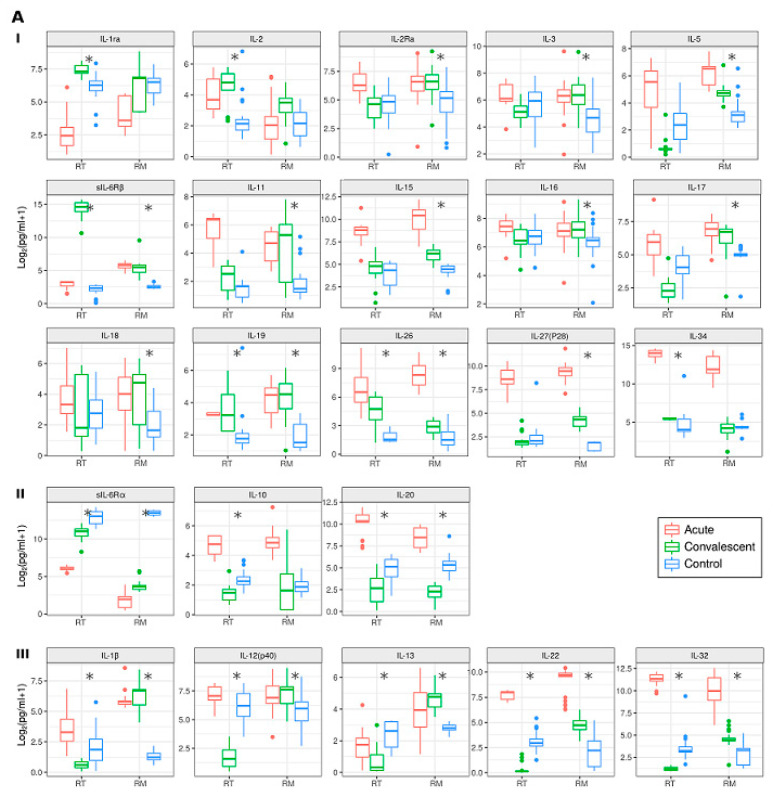

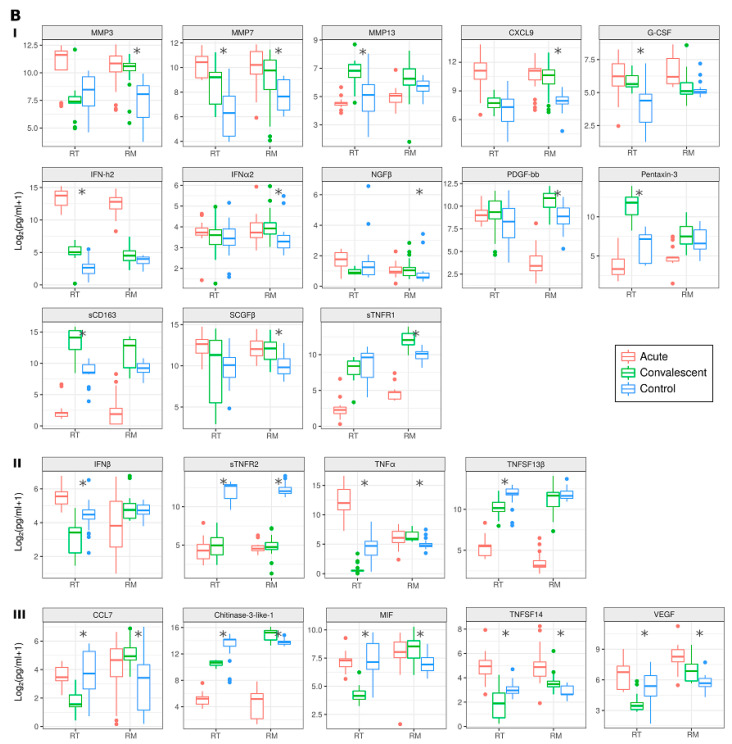
Analysis of levels of cytokines and MMPs in serum of the RT and the RM in acute and convalescent NE cases and controls. Panel (**A**) represent levels of interleukins, panel (**B**)—MMPs and cytokines; (**I**)—level of analytes is elevated in convalescent serum as compared to regional control in the RM and the RT; (**II**)—level of analytes is lower in convalescent serum as compared to regional control in the RM and the RT; (**III**)—changes in analyte levels in convalescent serum differ in the RM and the RT as compared to regional control. Asterisks indicate statistically significant differences between cytokines levels of convalescent patients and controls (*p* < 0.05, Kruskal–Wallis test with Benjamini–Hochberg adjustment).

Analysis of cytokine and MMP levels in acute vs. convalescent phases of NE. Serum cytokines and MMPs from the acute and convalescent phases of all NE were also analyzed (Figure 4: summarized in Supplementary Materials Tables S3 and S4).

Of the total number of cytokines and MMPs analyzed, there were 14 which were elevated during the acute phase of NE that returned to similar levels to controls during the convalescent phase (MMP1, MMP2, MMP8, MMP9, MMP12, TNFRSF8, IL-12(p70), IFN-h1, osteopontin, IL-4, IL-7, CCL2, CCL3, and CCL4) (Supplementary Materials Tables S3 and S4). However, we found that levels of IL-19, IL-26, sIL-6Rβ (Figure 4A), and MMP7 (Figure 4B) remained significantly higher in convalescent patients with NE from the RT and the RM and did not return to the basal level of regional controls. We also identified interleukins (sIL-6Rα, IL-20 (Figure 4A) and sTNFR2 (Figure 4B)) that were lower in convalescent NE serum of both the RT and the RM compared to that of regional controls. Notably, IL-20 levels were elevated during the acute phase of NE in both regions but decreased to lower levels than that of controls during the convalescent phase (Figure 4A).

Three interleukins (IL-1Ra, IL-2, IL-34 (Figure 4(AI)) and five cytokines and MMPs (IFN-h2, G-CSF, MMP13, Pentaxin-3, and sCD163 (Figure 4(BI)) were higher only in the convalescent phase of patients from the RT. While more analytes, nine interleukins (IL-2Ra, IL-3, IL-5, IL-11, IL-15, IL-16, IL-17, IL-18, IL-27(p28) (Figure 4(AI)), and seven cytokines and MMPs (MMP3, CXCL9, IFNα2, NGFβ, SCGFβ, PDGFbb, and sTNFR1 (Figure 4(BI)) remained higher during the convalescent phase of patients from the RM compared to those of regional controls.

We also identified IL-10 (Figure 4(AII)) as well as IFNβ and TNFSF13β (Figure 4(BII)) that had lower levels during convalescent phases in the RT. Interestingly levels of five interleukins (IL-1β, IL-12(p40), IL-13, IL-22, IL-32 (Figure 4(AIII)) and six cytokines (CCL7, Chitinase 3-like-1, MIF, TNFα, TNFSF14, and VEGF (Figure 4(BIII)) were lower during convalescent phases in the RT, whilst corresponding samples from the RM remained higher than regional controls.

**Figure 5:**
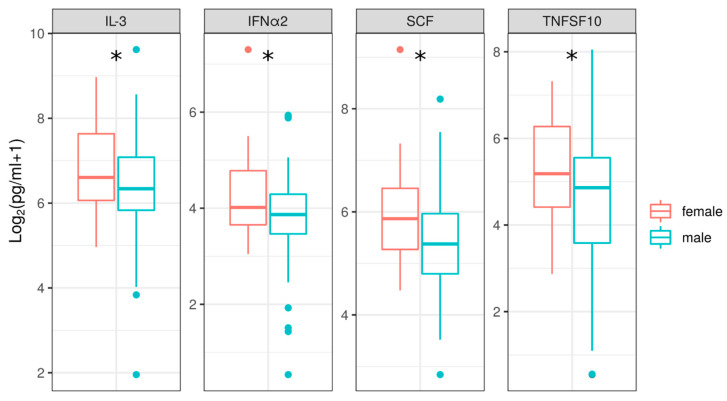
Analysis of serum cytokines of male and female patients with NE. Serum levels of cytokines were compared between the RM and the RT pooled female and male samples. Asterisks indicate statistically significant differences between cytokines levels of males and females (*p* < 0.05, Kruskal–Wallis test).

Analysis of cytokines in male and female NE. NE/HFRS have a strong bias towards more male patients than female patients [19,47]. However, it appears that the severity of disease [48] and the mortality rate in the first year following recovery is higher in females when compared to males [19]. Therefore, we sought to determine whether cytokine levels differ in male and female patients with NE. We examined acute samples from female and male patients from the RM and the RT to analyze serum cytokine level in each sex group. We observed significantly increased serum levels of IL-3, IFNα2, SCF, and TRAIL (TNFSF10) in female patients when compared to those of male patients (Figure 5).

## 4. Discussion

Analysis of clinical data of patients with NE from the RM and the RT revealed that the duration of the hospitalization period and febrile phase is longer in the RM compared to that in the RT. This prompted our investigation of orthohantavirus strains associated with NE in these regions. Many clinics throughout Russia use standard operating procedures (SOPs) for the treatment of patients presenting with HFRS or NE, suggesting that the differences in clinical presentation is not due to differences in treatment methods between regions [49]. Orthohantavirus RNA in serum samples was analyzed using PCR primers for PUUV, DOBV, TULV; however, PCR products were only generated when using PUUV primers. Therefore, these data suggest that PUUV is the main causative agent of NE in the RM and the RT and suggest that PUUV lineage-related differences are not the dominant factor contributing to the differences in clinical presentation of NE. Therefore, we conclude that extended hospitalization and prolonged febrile phase in patients with NE in the RM compared to that in the RT is more likely due to local reactivity to orthohantavirus infection and is unrelated to PUUV lineage. To test this hypothesis, we observed differential levels of several serum cytokines in controls from the RM and the RT. This observation suggests that the immune reactivity of the local population in general differs between the RM and the RT regions even in the absence of PUUV infection. Multiple factors could affect the levels of serum cytokines and MMPs, including regional dietary preferences, lifestyle, and environmental factors.

Hantavirus pathogenesis can be explained by the “cytokine storm” hypothesis, where clinical symptoms are the result of overproduction of proinflammatory cytokines [22,50,51]. Therefore, we sought to determine whether expression of serum cytokines in patients with NE differed between regions of the RT and the RM. NE cases from both the RM and the RT were characterized by upregulation of proinflammatory cytokines, as similarly reported in previous studies [31,32]. However, it appears that the magnitude of upregulation of several cytokines was higher in patients with NE from the RM compared to that of patients with NE from the RT. The most striking observation was an upregulation of several MMPs in the serum of patients with NE from the RT and the RM compared to that of local controls. MMP7, 8, 9, 12, and 13 are tightly regulated and prolonged periods of elevated MMP levels in serum can lead to increased immunopathology and prolonged clinical presentation [52,53]. Our data also demonstrate the upregulation of MMP9 in NE cases from the RT and the RM. Interestingly, studies have shown that Th1 lymphocytes producing MMP9 have higher migratory capacities in comparison to that of Th2 lymphocytes [54], suggesting that this MMP could contribute to the role of leukocyte subsets in the pathogenesis of NE. Hence, MMPs may contribute to the pathogenesis of NE in the RM and the RT [55].

We found increased levels of several cytokines in the sera of patients with NE from the RM compared to that of patients with NE from the RT. For example, we have found that several chemokines such as CCL2, CCL3, and CCL4 were higher in patients with NE from the RT compared to that of patients with NE from the RM. In addition, cytokines with strong inflammatory activity, IL-1α and IL-1β, were higher in patients with NE from the RM compared to that of patients with NE from the RT. In contrast, anti-viral IFNα2 and IFNβ were higher in patients with NE from the RT compared to that of patients with NE from the RM, but that the period of hospitalization and duration of fever of patients with NE was shorter in the RT when compared to that of the RM. These IFNs play a critical and central role in the innate immune response to viral infection [56]. These data suggest the possible connection between higher IFN production and milder clinical symptoms in NE.

Amongst the upregulated interleukins, the following families could be identified: the IL-1 (IL-1β and IL-18), common γ chain cytokine (IL-2, IL-2Ra, IL-4, IL-7 and IL-9), IL-10 (IL-26), and IL-12 (IL-12p70, IL-12p40, IL-27p28). The IL-1β and IL-18, members of the IL-1 family, have strong pro-inflammatory functions, responsible for many symptoms of inflammation [57,58,59]. A combined effect of the IL-1 and IL-12 families of cytokines could support proliferation of Th1 lymphocytes [60], thereby playing a role in antiviral defense [61]. In addition to Th1 lymphocytes, activation of Th2 immune responses is also evident in Patients with NE from the RM, where increased levels of IL-4, IL-5, and IL-9 are detected [62]. Strong evidence supporting the activation of NK cells in the RM NE is demonstrated by increased serum levels of IL-3, IL-5, and IL-9 [63]. These data suggest that enhanced inflammatory responses and higher activation of Th1 and Th2 types of the immune response combined with NK activation contribute to differences in clinical presentation of NE in the RM in comparison to that in the RT.

Interestingly, the level of IFN-γ in the sera of patients with NE was higher in the RM when compared to that in the RT. IFN-γ is produced only by limited subset of leukocytes, such as activated CD8+ T lymphocytes, γδT cells, and natural killer (NK) cells [55,64,65], thus contributing to sustained paracrine and autocrine activation [66]. Under physiological conditions, IFN-γ contributes to leukocyte function, control of cell proliferation, apoptosis, and cytokine secretion [67,68,69]. However, in a “cytokine storm” environment, IFN-γ could synergize with pro-inflammatory cytokines, triggering cell death, tissue damage, and fatal cytokine shock [70,71]. We suggest that higher level of IFN-γ in the RM NE could contribute to differences in the clinical presentation of the disease in these two regions of the Russian Federation.

An increased serum level of IL-3, IFNα2, SCF, and TRAIL (TNFSF10) in patients with NE was found in females when compared to that of males. IL-3 and SCF contribute to proliferation and differentiation of stem cells, suggesting that proliferation of progenitors in patients with NE is more pronounced in females when compared to males [72,73]. Interestingly, in females these “proliferation promoting” cytokines were upregulated together with TRAIL. TRAIL is pro-apoptotic cytokine [74] that can be induced by IFNα [75] and is also found to be upregulated in females with NE when compared to males with NE. It has been suggested that TRAIL induction by virus infection could lead to apoptosis of infected cells [76]. We therefore propose that a faster clearance of virus occurs in females compared to that of males but highlight that the protective role of TRAIL could lead to enhanced cell death and tissue damage. Further studies will help to determine the role of TRAIL in higher severity [48] and mortality rates in the first year after recovery in females versus males [19].

In conclusion, we have shown that NE cases in the RT and the RM are associated with PUUV infection. Clinical symptoms of NE in both locations were similar; however, the hospitalization and duration of the febrile phase was longer in patients with NE from the RM than those from the RT. We have shown elevated levels of several serum cytokines, chemokines, and MMPs in patients with NE from the RT and the RM, thus contributing to the cytokine storm hypothesis of NE pathogenesis and suggesting the occurrence of leukocyte activation, extracellular matrix degradation, and leukocyte chemotaxis. However, several cytokines were differentially expressed in NE serum between the two regions, which may contribute to differences in the clinical presentation of NE between the two regions. These differences are not related to the genetic variation of orthohantaviruses circulating in those regions as only the RUS lineage of PUUV was detected. Therefore, we conclude that demonstrated differences in serum cytokine levels between patients with NE from the RT and those of the RM are related to individual host immune responses to infection and hypothesize that these responses are influenced by multiple regional, environmental, and host factors. Identification of these factors could lead to improved and more personalized management protocols for patients with NE.

## Figures and Tables

**Table 1:** Primers used for amplification and sequencing.

Name	Nested-PCR Step	Sequence, 5′→3′	Binding Position ^c^	Reference
PUUV-39S-F3 ^a,b^	1st	GGCCAAAACATCTATATGTATCC	560–582 ^d^	
PUUV-S-R1496 ^a,b^	1st	GTATAATTCCAGTTAACCCCTG	1496–1517 ^d^	[33]
PUUV-S-F704 ^a,b^	2nd	AACATCATGAGTCCAGTAATGGG	682–704 ^d^	[33]
PUUV-69S-B3 ^a,b^	2nd	GATATCTCTTTTACCTTCTGGTC	1297–1319 ^d^	[33]
DOBV-F212 ^a^	1st	GAAAAGAAAGGGATCCAACTGG	191–212 ^e^	
DOBV-R542 ^a^	1st	ATACTGGATTGTGCATTGGGC	542–562 ^e^	
DOBV-F348 ^a^	2nd	ATGAACCAACAGGGCAAACTG	327–348 ^e^	
DOBV-R518 ^a^	2nd	GACAGAAACAGGTGCTTTGGC	518–538 ^e^	
TulaV-For49 ^a^	1st	AAGGATCCTCTAGAAACCGCTGGTATGAGCC	19–49 ^f^	
TulaV-Rev1321 ^a^	1st	GTGTCTGCAGGATCCGTTGATTAGATTTTTAGTGG	1321–1355 ^f^	
TulaV-For91 ^a^	2nd	AGATCACCCGCCATGAACAGC	71–91 ^f^	
TulaV-Rev328 ^a^	2nd	CATCAAGGACATTCCCATATCTGAG	328–352 ^f^	

^a^—Primers used for amplification; ^b^—primers used for sequencing; ^c^—positions in relation to: ^d^—Puumala orthohantavirus strain Puu/Kazan (GenBank accession no. Z84204); ^e^—Dobrava-Belgrade orthohantavirus isolate Ap-1/Goryachiy Klyuch-2000 (GenBank accession no. AF442622); ^f^—Tula orthohantavirus strain (Tula/76Ma/87) (GenBank accession no. Z30941).

**Table 2:** Clinical and laboratory characteristics of patients with NE from the RT and the RM.

	RT	RM	*p*-Value
Age (years)	37.50 (29.75, 51.50); *n* = 96	33.50 (26.25, 48.75) *n* = 58	0.203
Hospitalization (days)	11.00 (8.00, 12.00); *n* = 72	16.00 (14.25, 16.00); *n* = 58	<0.001
Fever (days)	6.00 (4.00, 7.00) *n* = 54	8.00 (7.00, 9.00); *n* = 58	<0.001
Duration of hemorrhage (days)	0.00 (0.00, 0.00); *n* = 92	0.00 (0.00, 0.00); *n* = 58	0.641
Systolic arterial pressure (mmHg)	116.00 (110.00, 120.00); *n* = 55	120.00 (110.00, 137.50); *n* = 58	0.120
Diastolic arterial pressure (mmHg)	80.00 (70.00, 80.00); *n* = 55	80.00 (70.00, 90.00); *n* = 58	0.065
Urea (mg/dL)	7.10 (4.70, 12.60); *n* = 93	6.65 (4.85, 10.25); *n* = 58	0.750
Creatinine (µM/L)	131.00 (103.00, 189.00); *n* = 93	121.50 (96.00, 160.50); *n* = 58	0.172
Potassium (mEq/L)	7.10 (5.39, 10.75); *n* = 88	5.76 (3.95, 8.07); *n* = 58	0.004
Thrombocytes (×10^3^/µL)	92.00 (67.00, 157.00); *n* = 93	78.50 (58.50, 109.75); *n* = 58	0.205
Leukocytes (×10^3^/µL)	9.30 (6.20, 13.65); *n* = 55	7.50 (6.62, 9.73); *n* = 58	0.251
vRNA +/− (+%/−%)	72/25 (74.2/25.8); *n* = 96	10/7 (58.8/41.2); *n* = 17	0.242
Hantavirus IgM +/− (+%/−%)	75/96 (78.1/21.9); *n* = 96	33/58 (56.9/43.1); *n* = 58	0.711
Hantavirus IgG +/− (+%/−%)	86/96 (86.4/13.6); *n* = 96	50/8 (86.2/13.8); *n* = 58	0.096

## Data Availability

Not applicable.

## Supplementary Materials

The following are available online at https://www.mdpi.com/article/10.3390/pathogens10050527/s1, Table S1: Analysis of acute serum cytokine and MMPs levels in RT and RM controls; Table S2: Analysis of serum cytokine and MMPs levels in RT and RM acute patients; Table S3: Analysis of acute serum cytokines and MMPs level in NE from RT; **Table S4**: Analysis of cytokines and MMPs in serum of RM NE cases in acute and convalescent stages.
