# Supplementary material for: Cytokine, Chemokine, and Metalloprotease Activation in the Serum of Patients with Nephropathia Epidemica from the Republic of Tatarstan and the Republic of Mordovia, Russia
Source: Pathogens. 2021 Apr 27;10(5):527. doi: 10.3390/pathogens10050527 (PMC8145562; doi:10.3390/pathogens10050527)
Supplement: Supplementary file 1 [file pathogens-10-00527-s001.zip › pathogens-1162779-supplementary.pdf]

Supplementary Table 1. Analysis of acute serum cytokine and MMPs levels in RT and RM controls

Cytokine (pg/mL)	RT control (n-27)	RM control (n -19)	P. adjust
IL-1α	0.91±0.86	0.64±1.4	0.02
IL-1β	5.59±10.42	1.57±0.82	
IL-1ra	84.21±46.27	85.95±47.2	
IL-2Ra	31.72±25.19	47.33737±57	
IL-2	8.58±21.08	4.54±3.52	
IL-3	70.49±54.15	45.02±57.15	
IL-4	4.51±3.15	5.88±0.99	
IL-5	7.15±9.19	13.93±20.59	
IL-6	40.46±68.09	24.04±5.6	0.04
sIL-6Ra	9226.61±4935.31	11644.65±2489.66	
sIL-6Rb	3.8 ±2.05	5.12±1.39	
IL-7	8.73±12.02	14.64±7.45	0.03
IL-8	28.67±21.92	22.23±19.46	
IL-9	37.8±33.36	57.98±33.69	
IL-10	4.47±2.63	3.22±1.95	
IL-11	2.23±2.92	5.28±8.6	
IL-12(p70)	53.12±97.25	44.56±20.49	
IL-12(p40)	105.01±81.16	89.59±109.57	
IL-13	4.88±2.93	6.13±1.24	
IL-15	20.2±14.55	20.6±8.58	

IL-17	19.1±13.18	31.68±9.48	
IL-16	111.21±61.26	103.53±79.13	
IL-18	8.35±9.1	4.23±4.68	
IL-19	8.67±31.78	3.3±2.64	
IL-20	40.09±26.08	57.85±83.75	
IL-22	9.37±8.33	6.65±9.23	
IL-26	2.8±1.63	3.75±4.84	
IL-27(p28)	14.52±55.69	2.043±0.93	
IL-32	35.06±127.7	8.66±8.28	
IL-34	101.78±395.27	21.35±12.67	
IL-35	27.96±26.87	22.07±18.33	
CCL2	44.87±97.65	25.15±17.57	
CCL3	9.97±13.34	4.57±1.55	
CCL4	83.98±109.86	68.31±42.84	
CCL5	190.37±219.53	149.05±99.39	
CCL7	19.56±18.7	16.21±28.52	
CCL11	127.78±166.58	139.9±53.17	
CCL27	80.71±45.97	61.52±43.22	
CXCL1	39.74±30.52	28.36±20.01	
CXCL9	229.16±259.92	266.03±138.96	
CXCL10	181.54±176.06	218.47±116.73	
CXCL12	55.5±45.13	44.67±56.83	

Chitinase-3-like1	14526.85±8536.4	14972.82±5142.39	
CSF	34.02±17.09	31.73±18.21	
FGFbasic	17.51±17.71	19.56±8.97	
G-CSF	25.97±31.11	41.53±28.27	
GM-CSF	12.07±15.21	18.58±5.7	0.002
HGF	135.05±130.9	105.01±115.09	
LIF	4.2±3.54	6.29±7.79	
M-CSF	2.55±1.68	3.7±4.26	
MIF	264.73±255.08	149.44±103.21	
b-NGF	5.33±17.86	1.3±2.44	0.02
SCGFb	1791.85±2224.11	1794.47±1962.12	
TNFα	42.67±85.26	40.2±39.05	
TNFβ	1.49±3.72	1.12±2.14	
MMP1	69.98±78.9	109.47±112.4	
MMP2	130.7±114.3	149.65±122.2	
MMP3	459.7±367.6	349.75±315.03	
MMP7	186.88±272.6	280.81±222.95	
MMP8	47.35±50.5	12.82±11.76	
MMP9	1528.8±2417.2	2079.49±1165.22	
MMP10	57.6±53.5	94.88±119.02	
MMP12	380.87±552.25	53.13±19.16	

MMP13	48.74±54.3	531.47±408.6	0.03
sCD163	583.95±434.97	724.96±461.65	
Osteocalcin	1019.96±1215.6	1032.61±478.47	
Osteopontin	8423.1±4494.06	6695.9±3856.69	
PDGF-bb	577.6±774.39	619.6479±527.23	
Pentaxin-3	129.82±125.05	207.57±209.65	
sTNFR1	780.56±657.19	1098.69±593.68	
sTNFR2	5260.21±2924.97	5866.91±4089.69	
TNFRSF8	838.08±1278.79	816.89±738.93	
TNFSF10	24.14±14.14	31.69±22.63	
TNFSF13	51382.64±165254.05	13490.18±18639.03	
TNFSF13b	4151.3±1937.29	4343.57±2830.39	
TNFSF12	58.25±75.37	52.52±42.82	
TNFSF14	8.01±4.35	6.7±2.39	
TSLP	650.35±3265.11	20.23±9.58	
VEGF	57.49±55.24	59.99±41.2	
IFN-α2	11.31±6.61	11.73±9.99	
IFNβ	23.2±15.78	26.06±7.77	
IFNγ	41.89±39.56	23.4±21.44	
IFN-h2	8.77±11.06	13.66±6.02	
IFN-h1	10±10.13	14.86±13.26	

P. adjust – Benjamini-Hochberg adjusted p-value.

Supplementary Table 2. Analysis of serum cytokine and MMPs levels in RT and RM acute patients

Cytokine (pg/mL)	RT acute (n-98)	RM acute (n-25)	P. adjust
IL-1α	0.35±0.79	0.42±0.39	0.003
IL-1β	26.49±72.76	66.92±65.26	<0.0001
IL-1ra	8.13±11.25	24.07±18.25	0.0005
IL-2Ra	125.83±125.26	139.00±152.76	
IL-2	20.18±16.58	6.74±9.99	<0.0001
IL-3	129.38±101.37	108.47±147.82	
IL-4	19.23±28.63	51.06±33.49	<0.0001
IL-5	61.53±76.82	84.68±45.80	0.006
IL-6	18.32±31.59	92.41±168.67	<0.0001
sIL-6Ra	63.54±14.97	3.12±3.11	<0.0001
sIL-6Rb	6.76±8.77	54.14±18.82	<0.0001
IL-7	48.72±105.56	57.60±47.22	0.048
IL-8	47.23±36.84	14.68±13.96	<0.0001
IL-9	88.65±95.62	231.06±155.41	<0.0001
IL-10	38.56±25.27	35.19±27.70	
IL-11	38.37±33.05	28.21±18.51	
IL-12(p70)	56.73±49.73	16.00±12.35	0.0001

IL-12(p40)	273.16±460.87	169.81±134.22	
IL-13	3.14±3.08	23.24±23.84	<0.0001
IL-15	814.48±1012.45	1525.43±1233.32	
IL-17	73.28±95.95	128.07±69.62	0.0001
IL-16	231.62±339.70	170.31±116.09	
IL-18	22.90±27.58	23.80±22.48	
IL-19	10.45±7.80	21.23±12.77	0.005
IL-20	1572.18±1356.02	475.41±320.87	0.006
IL-22	237.30±55.74	746.94±398.49	0.02
IL-26	343.55±484.72	496.67±440.54	
IL-27(p28)	665.58±641.69	821.18±676.19	
IL-32	2870.33±1603.01	1662.57±1626.24	
IL-34	15534.99±6220.72	7214.97±5564.08	0.004
IL-35	13.22±13.98	61.27±62.04	<0.0001
CCL2	162.72±149.97	1500.42±801.92	<0.0001
CCL3	561.81±952.47	1133.72±1123.01	0.04
CCL4	1133.73±1367.24	4353.96±3455.27	<0.0001
CCL5	370.40±874.51	324.46±166.02	0.0003
CCL7	21.60±35.83	28.89±23.91	
CCL11	351.22±963.23	186.47±168.42	

CCL27	51.06±34.37	44.33±29.21	
CXCL1	25.89±15.04	28.08±14.11	
CXCL9	2419.50±2419.54	2291.08±1923.97	
CXCL10	469.49±1132.36	130.65±145.70	
CXCL12	46.97±29.71	41.49±17.12	
Chitinase-3-like1	48.38±44.27	50.89±61.47	
CSF	60.79±64.20	45.20±36.59	
FGFbasic	41.53±58.24	160.70±185.43	<0.0001
G-CSF	101.87±111.34	129.13±100.79	0.04
GM-CSF	9.82±9.05	112.13±81.57	<0.0001
HGF	191.56±212.80	147.48±213.44	
LIF	7.55±6.62	5.88±4.78	
M-CSF	5.77±4.67	3.85±3.06	0.04
MIF	224.48±229.47	321.54±213.02	0.03
b-NGF	3.44±2.42	1.17±0.74	<0.0001
SCGFb	6094.12±5018.49	6314.80±5453.49	
TNFα	14734.16±25202.80	106.11±88.50	<0.0001
TNFβ	0.62±0.58	0.67±0.41	
MMP1	16329.36±14396.10	17474.39±20478.62	
MMP2	4963.80±2477.77	5304.73±3249.50	

MMP3	2360.78±1891.49	2016.27±1458.80	
MMP7	1397.76±2001.56	1516.23±1111.43	
MMP8	24413.25±9076.77	17264.39±5173.99	0.03
MMP9	42343.01±15616.73	49439.12±15009.58	
MMP10	32.34±34.31	37.73±57.40	
MMP12	11.93±16.85	30.24±16.60	0.0003
MMP13	23.83±11.28	33.15±19.68	0.02
sCD163	15.77±27.68	24.53±67.20	
Osteocalcin	4.02±3.66	11.53±5.85	0.0004
Osteopontin	5.65±4.77	12.42±6.54	0.01
PDGF-bb	965.43±872.96	27.59±54.91	<0.0001
Pentaxin-3	23.91±44.94	40.64±46.07	0.0026
sTNFR1	9.05±11.58	29.79±33.42	0.0006
sTNFR2	33.27±43.05	26.95±16.57	
TNFRSF8	21.43±12.52	8.37±10.13	0.0032
TNFSF10	45.05±47.38	34.05±28.72	
TNFSF13	14993.50±7231.82	14283.08±3079.13	
TNFSF13b	69.13±105.32	14.86±18.91	0.002
TNFSF12	55.39±112.54	170.31±157.54	<0.0001
TNFSF14	50.04±50.83	53.84±70.96	

TSLP	41.69±48.53	84.59±80.50	0.006
VEGF	217.45±295.58	422.86±458.97	0.002
IFN-α2	17.96±17.31	15.56±10.39	
IFNβ	46.90±27.82	26.22±26.95	0.0002
IFNγ	10.54±12.12	25.68±24.60	0.03
IFN-h2	15060.12±12582.63	9133.16±8437.95	
IFN-h1	307.04±239.56	2715.27±3784.50	0.006

P. adjust – Benjamini-Hochberg adjusted p-value.

Supplementary Table 3. Analysis of acute serum cytokines and MMPs level in NE from RT .

Cytokine (pg/mL)	acute (n=17)	control (n=27)	convalescent (n=17)	P adj. value acute - control	P adj. value acute - convalescent	P adj. value control -convalescent
IL-1α	0.21±0.26	0.92±0.86	1.00±3.26	0.001		
IL-1β	17.19±26.40	5.60±10.43	0.55±0.36		0.0001	0.02
IL-1ra	10.65±16.93	84.22±46.27	175.01±47.65	<0.0001	<0.0001	0.003
IL-2Ra	114.17±91.31	31.73±25.20	27.36±20.94	0.0003	0.0003	
IL-2	19.92±17.31	8.59±21.09	27.35±16.85	0.0002		<0.0001
IL-3	98.23±57.63	70.50±54.16	36.80±18.03		0.002	
IL-4	22.55±51.38	4.51±3.15	2.41±1.74	0.01	0.0003	
IL-5	55.93±51.71	7.16±9.20	0.97±1.77	0.0001	<0.0001	
IL-6	28.13±66.77	40.47±68.10	0.44±0.72		0.003	0.0001
sIL-6Ra	65.38±13.61	9226.62±4935.32	2023.61±1100.21	0.0003		0.03
sIL-6Rb	6.64±2.78	3.80±2.06	26331.78±16823.57		<0.0001	<0.0001
IL-7	41.36±50.31	8.73±12.03	5.00±2.53	<0.0001	<0.0001	
IL-8	45.01±33.75	28.68±21.92	21.77±54.51		<0.0001	0.001
IL-9	69.84±82.18	37.82±33.36	20.61±17.19		0.03	
IL-10	27.32±11.55	4.47±2.63	1.91±1.40	0.0001	<0.0001	0.01
IL-11	61.58±34.35	2.23±2.92	4.66±3.37	<0.0001	<0.0001	
IL-12(p70)	82.45±74.41	5.86±7.08	3.48±1.27	<0.0001	<0.0001	
IL-12(p40)	157.70±76.42	105.01±81.16	3.15±3.03		<0.0001	<0.0001
IL13	3.21±4.14	4.89±2.93	1.12±1.73			0.003

IL-15	515.28±523.05	20.29±14.56	30.01±26.17	<0.0001	<0.0001	
IL-17	89.76±127.63	19.11±13.19	5.60±5.62	0.002	<0.0001	
IL-16	185.37±78.88	111.21±61.27	101.47±53.49	0.01	0.01	
IL-18	27.93±38.47	8.36±9.11	18.77±21.89			
IL-19	8.65±0.49	8.67±31.78	15.36±16.01	0.001		0.0004
IL-20	1472.56±969.55	40.09±26.08	8.73±10.53	<0.0001	<0.0001	0.01
IL-22	224.22±60.65	9.38±8.33	0.42±0.75	<0.0001	<0.0001	0.005
IL-26	301.23±555.13	2.80±1.63	34.60±32.17	<0.0001	0.02	0.004
IL-27(p28)	518.48±353.32	14.53±55.69	4.05±3.99	<0.0001	<0.0001	
IL-32	2739.28±1102.19	35.06±127.71	1.39±0.37	<0.0001	<0.0001	0.003
IL-34	16003.23±5604.48	101.78±395.27	42.42±3.26	<0.0001	0.0003	0.03
IL-35	11.47±14.36	27.97±26.88	1.14±1.91		0.004	<0.0001
CCL2	144.74±106.02	44.87±97.65	25.26±24.66	0.0002	0.0001	
CCL3	261.64±232.33	9.98±13.35	4.09±2.73	<0.0001	<0.0001	
CCL4	897.60±1289.39	83.99±109.87	46.48±24.57	<0.0001	<0.0001	
CCL5	198.91±309.19	190.37±219.53	826.55±697.66		0.002	0.002
CCL7	11.64±5.71	19.56±18.70	2.92±2.27		0.03	0.002
CCL11	609.65±1996.51	127.78±166.59	46.82±36.97		0.003	
CCL27	49.49±28.02	80.72±45.97	69.52±60.58			
CXCL1	28.98±18.84	39.75±30.53	14.62±22.99		0.03	0.0002
CXCL9	3401.23±3693.71	229.16±259.92	230.04±130.66	<0.0001	<0.0001	

CXCL10	1208.14±2235.60	181.54±176.07	64.80±89.23		0.001	0.003
CXCL12	55.54±32.71	55.50±45.14	39.85±13.32			
Chitinase-3-like1	41.70±42.81	14526.85±8536.43	1559.90±407.88	<0.0001	0.02	0.01
CSF	47.91±22.02	34.03±17.10	44.64±18.98			
FGFbasic	43.95±88.20	17.52±17.72	16.62±9.60			
G-CSF	105.14±89.73	25.97±31.12	64.70±30.59	<0.0001		<0.0001
GM-CSF	9.42±5.41	12.07±15.22	0.23±0.40		0.01	0.002
HGF	163.84±185.06	135.06±130.91	106.55±111.77			
LIF	8.50±4.27	4.22±3.55	2.52±1.71	0.02	0.002	
M-CSF	6.11±2.67	2.56±1.69	3.00±6.38	0.003	0.0002	
MIF	167.35±130.09	264.73±255.08	21.10±15.14		0.001	<0.0001
b-NGF	2.53±1.30	5.34±17.87	0.95±0.23		0.01	
SCGFb	8262.76±7717.84	1791.85±2224.12	5179.22±6367.81	0.0001		
TNFα	15426.96±24198.13	42.67±85.27	1.23±2.31			0.003
TNFβ	0.48±0.57	1.49±3.73	0.72±0.80			
MMP1	12461.17±7411.01	69.98±78.90	315.09±402.21	<0.0001	<0.0001	
MMP2	5425.89±3239.72	130.71±114.39	3994.87±15497.14	<0.0001	<0.0001	
MMP3	2791.32±1690.96	459.78±367.68	414.57±1027.84	<0.0001	<0.0001	
MMP7	1543.11±1094.84	186.89±272.60	728.11±719.54	<0.0001		0.01
MMP8	22976.60±6847.29	47.35±50.55	356.24±1251.79	<0.0001	<0.0001	
MMP9	39842.37±16035.21	1528.85±2417.29	1267.21±1742.96	<0.0001	<0.0001	

MMP10	25.74±28.84	57.65±53.53	366.77±354.05		<0.0001	<0.0001
MMP12	7.45±9.13	380.88±552.26	223.59±186.23	<0.0001	<0.0001	
MMP13	23.20±8.07	48.74±54.33	124.43±87.75	0.04	<0.0001	0.0001
sCD163	13.05±28.98	583.95±434.97	21711.96±19298.08	<0.0001	<0.0001	0.001
Osteocalcin	4.07±3.50	1019.96±1215.61	2387.15±1937.42	<0.0001	<0.0001	0.05
Osteopontin	4.84±3.72	8423.10±4494.07	6752.83±5956.84	<0.0001	<0.0001	
PDGF-bb	672.90±515.06	577.65±774.39	983.43±948.23			
Pentaxin-3	23.50±37.96	129.83±125.05	4734.17±4238.92	0.004	<0.0001	<0.0001
sTNFR1	9.65±22.75	780.57±657.20	373.88±228.82	<0.0001	0.0002	
sTNFR2	38.35±56.63	5260.21±2924.98	52.02±63.56	<0.0001		<0.0001
TNFRSF8	20.08±6.28	838.08±1278.79	402.64±282.84	<0.0001	0.002	
TNFSF10	27.04±19.56	24.14±14.15	6.84±6.74		0.002	0.001
TNFSF12	24.30±24.88	58.26±75.37	1.89±2.37		0.01	<0.0001
TNFSF13	13232.11±7613.66	51382.64±165254.05	78124.93±122492.68			
TNFSF13b	59.95±75.15	4151.32±1937.30	1580.07±1279.25	<0.0001	0.02	0.01
TNFSF14	42.08±54.54	8.01±4.35	4.14±4.53	<0.0001	<0.0001	0.04
TSLP	52.23±55.29	650.35±3265.11	5.77±6.74		<0.0001	0.001
VEGF	138.71±140.96	57.49±55.25	15.26±14.52	0.03	<0.0001	0.02
IFNα2	13.63±5.56	11.31±6.61	12.14±7.75			
IFNβ	48.94±20.76	23.20±15.78	10.69±8.71	0.0001	<0.0001	0.02
IFNγ	14.87±16.90	18.80±41.68	12.93±7.81			

IFN-h2	356.81±167.06	10.00±10.14	20.17±22.45	<0.0001	0.0003	
IFN-h1	14110.19±11048.41	8.77±11.06	41.53±28.87	<0.0001	0.001	0.001

P. adjust – Benjamini-Hochberg adjusted p-value.

Supplementary table 4. Analysis of cytokines and MMPs in serum of RM NE cases in acute and convalescent stages.

Cytokine (pg/mL)	acute (n=25)	control (n=19)	convalescent (n=25)	P. adjust acute - control	P. adjust acute - convalescent	P. adjust control -convalescent
IL-1α	0.42±0.39	0.65±1.39	0.65±1.40			
IL-1β	66.92±65.26	1.57±0.82	102.07±77.59	<0.0001		<0.0001
IL-1ra	24.07±18.25	85.95±47.20	114.23±129.28	0.001	0.001	
IL-2Ra	139.00±152.76	47.34±57.00	122.29±116.57	0.002		0.001
IL-2	6.74±9.99	4.54±3.52	9.90±5.53		0.004	0.01
IL-3	108.47±147.82	45.02±57.15	116.35±144.88	0.002		0.002
IL-4	51.06±33.49	5.88±0.99	13.33±8.99	<0.0001	0.001	
IL-5	84.68±45.80	13.93±20.59	28.51±17.94	<0.0001	0.01	0.02
IL-6	92.41±168.67	24.04±5.61	31.12±20.72			
sIL-6Ra	3.12±3.11	11644.65±2489.66	14.10±10.70	<0.0001	0.04	<0.0001
sIL-6Rb	54.14±18.82	5.12±1.39	68.60±142.68	<0.0001		<0.0001
IL-7	57.60±47.22	14.64±7.45	17.74±3.31	0.001		
IL-8	14.68±13.96	22.23±19.46	11.55±18.84			0.01
IL-9	231.06±155.41	57.98±33.69	131.40±138.00	0.0002	0.02	
IL-10	35.19±27.70	3.22±1.95	6.15±11.36	<0.0001	<0.0001	
IL-11	28.21±18.51	5.28±8.61	49.30±58.33	0.0001		0.001
IL-12(p70)	16.00±12.35	2.74±2.46	2.15±1.99	<0.0001	<0.0001	
IL-12(p40)	169.81±134.22	89.59±109.57	197.19±149.22	0.02		0.01
IL13	23.24±23.84	6.14±1.24	27.26±14.13	0.02		0.0003

IL-15	1525.43±1233.32	20.60±8.58	70.52±32.69	<0.0001	0.0002	0.0003
IL-17	128.07±69.62	31.68±9.48	89.45±40.36	0.0001		0.003
IL-16	170.31±116.09	103.53±79.14	185.96±148.62	0.02		0.02
IL-18	23.80±22.48	4.23±4.68	26.42±23.66	0.01		0.004
IL-19	21.23±12.77	3.30±2.64	25.92±17.54	<0.0001		<0.0001
IL-20	475.41±320.87	57.85±83.75	4.33±2.82	0.002	<0.0001	0.0003
IL-22	746.94±398.49	6.65±9.23	28.63±15.63	<0.0001	0.0002	0.003
IL-26	496.67±440.54	3.75±4.84	7.07±3.78	<0.0001	<0.0001	0.02
IL-27(p28)	821.18±676.19	2.04±0.93	19.65±11.05	<0.0001	0.001	<0.0001
IL-32	1662.57±1626.24	8.66±8.28	27.30±19.20	<0.0001	0.002	0.01
IL-34	7214.97±5564.08	21.35±12.67	20.89±14.13	<0.0001	<0.0001	
IL-35	61.27±62.04	22.07±18.33	15.08±18.09		0.002	
CCL2	1500.42±801.92	25.15±17.57	35.29±15.54	<0.0001	<0.0001	
CCL3	1133.72±1123.01	4.57±1.55	7.31±6.18	<0.0001	<0.0001	
CCL4	4353.96±3455.27	68.31±42.84	117.06±69.14	<0.0001	<0.0001	
CCL5	324.46±166.02	149.06±99.39	241.95±331.14	0.02	0.02	
CCL7	28.89±23.91	16.21±28.52	39.63±27.25	0.02		0.0001
CCL11	186.47±168.42	139.90±53.17	97.04±165.78		0.01	0.01
CCL27	44.33±29.21	61.52±43.22	44.18±33.94			
CXCL1	28.08±14.11	28.36±20.01	43.70±34.41			
CXCL9	2291.08±1923.97	266.03±138.96	1900.74±1775.93	0.0001		0.001

CXCL10	130.65±145.70	218.47±116.74	37.31±26.93		0.003	0.00001
CXCL12	41.49±17.12	44.69±56.83	47.96±30.72			
Chitinase-3-like1	50.89±61.47	14972.82±5142.39	35152.95±17378.32	<0.0001	<0.0001	0.04
CSF	45.20±36.59	31.73±18.21	47.75±37.23			
FGFbasic	160.70±185.43	19.56±8.97	35.05±25.50	<0.0001	0.01	
G-CSF	129.13±100.79	41.53±28.27	55.88±73.33	0.0002	0.0003	
GM-CSF	112.13±81.57	18.58±5.70	28.06±20.78	0.001	0.003	
HGF	147.48±213.44	105.01±115.09	147.37±105.14			
LIF	5.88±4.78	6.29±7.80	7.76±6.90			
M-CSF	3.85±3.06	3.70±4.26	4.21±2.96			
MIF	321.54±213.02	149.44±103.21	426.54±324.95	0.03		0.01
b-NGF	1.17±0.74	1.33±2.44	1.40±1.31	0.02		0.02
SCGFb	6314.80±5453.49	1794.47±1962.12	5913.39±5584.19	0.001		0.002
TNFα	106.11±88.50	40.24±39.06	96.73±61.84	0.01		0.01
TNFβ	0.67±0.41	1.12±2.14	0.87±1.28			
MMP1	17474.39±20478.62	109.47±112.44	343.47±250.74	<0.0001	<0.0001	
MMP2	5304.73±3249.50	149.65±122.24	138.02±140.69	<0.0001	<0.0001	
MMP3	2016.27±1458.80	349.75±315.03	1493.00±777.72	<0.0001		0.0002
MMP7	1516.23±1111.43	280.81±222.95	1001.54±787.44	0.0002		0.01
MMP8	17264.39±5173.99	12.83±11.76	51.68±113.20	<0.0001	<0.0001	
MMP9	49439.12±15009.58	2079.50±1165.23	3409.97±3945.15	<0.0001	<0.0001	

MMP10	37.73±57.40	94.88±119.02	188.57±149.13		<0.0001	0.001
MMP12	30.24±16.60	531.47±408.61	288.80±181.70	<0.0001	<0.0001	
MMP13	33.15±19.68	53.13±19.16	96.59±71.31	0.03	0.0001	
sCD163	24.53±67.20	724.96±461.65	7514.74±7090.63	<0.0001	<0.0001	
Osteocalcin	11.53±5.85	1032.61±478.47	5852.06±8744.62	<0.0001	<0.0001	
Osteopontin	12.42±6.54	6695.92±3856.69	5095.55±10445.01	<0.0001	0.0001	
PDGF-bb	27.59±54.91	619.65±527.23	2053.87±1291.68	<0.0001	<0.0001	0.003
Pentaxin-3	40.64±46.07	207.58±209.65	306.77±316.05	0.01	0.0001	
sTNFR1	29.79±33.42	1098.69±593.68	5758.27±4055.51	<0.0001	<0.0001	0.01
sTNFR2	26.95±16.57	5866.91±4089.70	38.22±35.78	<0.0001		<0.0001
TNFRSF8	8.37±10.13	816.89±738.93	1316.35±1102.28	<0.0001	<0.0001	
TNFSF10	34.05±28.72	31.67±22.63	39.43±35.42			
TNFSF12	170.31±157.54	52.52±42.82	62.42±53.61	0.01	0.02	
TNFSF13	14283.08±3079.13	13490.18±18639.03	15272.31±23016.26			
TNFSF13b	14.86±18.91	4343.57±2830.40	3720.91±3770.34	<0.0001	<0.0001	
TNFSF14	53.84±70.96	6.70±2.39	13.55±12.82	<0.0001	0.01	0.01
TSLP	84.59±80.50	20.23±9.58	53.11±61.38	0.001		
VEGF	422.86±458.97	59.99±41.20	150.89±144.90	<0.0001	0.02	0.02
IFN-α2	15.56±10.39	11.73±9.99	17.16±11.37	0.03		0.01
IFNβ	26.22±26.95	26.07±7.78	31.90±22.87			
IFNγ	25.68±24.60	20.45±8.74	16.52±12.92			

IFN-h1	2715.27±3784.50	14.86±13.26	5.43±3.64	<0.0001	<0.0001	
IFN-h2	9133.16±8437.95	13.66±6.02	30.76±33.57	<0.0001	<0.0001	

P. adjust – Benjamini-Hochberg adjusted p-value.
